# Fabrication and Properties of Superhydrophobic Waterborne Polyurethane Composites with Micro-Rough Surface Structure Using Electrostatic Spraying

**DOI:** 10.3390/polym11111748

**Published:** 2019-10-24

**Authors:** Fangfang Wang, Lajun Feng, Guangzhao Li, Zhe Zhai, Huini Ma, Bo Deng, Shengchao Zhang

**Affiliations:** 1 School of Materials Science and Engineering, Xi’an University of Technology, Xi’an 710048, China; wff1170111008@163.com (F.W.); lgz0414@163.com (G.L.); zhaizhe1113@163.com (Z.Z.); 17789276539@163.com (H.M.); dengbo311@163.com (B.D.); 2 Key Laboratory of Corrosion and Protection of Shaanxi Province, Xi’an 710048, China; 3 Faculty of Printing, Packing Engineering and Digital Media Technology, Xi’an University of Technology, Xi’an 710048, China; zhangsc1364879367@163.com

**Keywords:** superhydrophobic WPU composites, electrostatic spraying, nano-SiO_2_ particles, adhesion, corrosion resistance, wear rate

## Abstract

Waterborne polyurethane (WPU) coatings hold advantages of good toughness, low cost and environmental protection. However, the low water contact angle (WCA), poor wear and corrosion resistance make them unsuitable for application in the superhydrophobic coatings such as antipollution flashover coatings for transmission lines, self-cleaning coatings for outdoor equipment and waterproof textiles. A series of superhydrophobic WPU composites (SHWPUCs) with micro-rough surface structure was prepared by electrostatic spraying nano-SiO_2_ particles on WPU composites with low surface energy. It showed that as the hydrophobic system content rose the WCAs of the composites first increased and then remained stationary; however, the adhesion and corrosion resistance first increased and then decreased. An appropriate addition of the hydrophobic system content would lead to a dense coating structure, but an excessive addition could increase the interfaces in the coating and then reduce the coating performance. When the mass ratio of the WPU dispersion, polytetrafluoroethylene (PTFE) particles and modified polydimethylsiloxane was 8:0.3:0.4, 10 g/m^2^ nano-SiO_2_ particles were sprayed on the uncured coating surface to construct the SHWPUC with a WCA of 156°. Compared with pure WPU coating, its adhesion and corrosion resistance increased by 12.5% and one order of magnitude, respectively; its wear rate decreased by 88.8%.

## 1. Introduction

Protective coatings with superhydrophobicity have a widespread application in the fields of antipollution flashover coatings applied in transmission lines, self-cleaning coatings applied in outdoor equipment and waterproof cloth, etc. [1,2,3]. However, the most widely used hydrophobic coatings are solvent-borne coatings and their volatile organic solvent (VOC) contents are commonly over 40 wt %. During the processes of production, storage and using, solvent-borne coatings are prone to emit a lot of organic solvents which can not only pollute the environment but also have some potential safety problems [4]. Additionally, these organic solvents are generally flammable and high cost. With the formulation of environmental laws and regulations around the world and the increasing environmental protection awareness of human nowadays, it would be inevitable for us to research and apply waterborne coatings holding advantages of low VOC emission, low toxicity and environmental protection. Waterborne polyurethane (WPU) coatings with water as the dispersion medium are basically low cost, solvent-free, non-toxic and non-flammable, in addition, they will not pollute the environment and can avoid safety problems during production and application compared with solvent-based PU coatings [5]. Furthermore, polar groups such as –COOH and –OH in the molecular chains of WPU can produce crosslinking polymerization reactions under certain conditions [6,7,8], thus, it could strengthen the coating structure and enhance the adhesion to the substrates. Due to these excellent properties WPU coatings are extensively applied in many industrial areas such as coatings, adhesives, synthetic leathers, packaging films, membranes, biomaterials and waterproof textiles [9,10,11,12].

However, the traditional WPU coatings have some drawbacks such as low water contact angle (WCA), poor wear resistance and corrosion resistance, so that their applications in the superhydrophobic coating are restricted [13]. In order to solve these problems, it is necessary to modify the WPU coating to improve its properties such as hydrophobicity and wear resistance and thus expand its application range [14]. Previous reports have shown that the surface hydrophobicity can be improved by the addition of low surface energy materials on rough surfaces or the construction of rough structures on low surface energy surfaces [15,16,17,18]. In recent years, researchers have improved the performances of WPU resins by adding materials containing elements such as fluorine and silicon. Polytetrafluoroethylene (PTFE) material holds excellent properties of high fluoride content, thermochemical stability, high hydrophobicity and low surface friction [19]. Additionally, the WCA of the untreated PTFE coating is up to 120° and the addition of PTFE materials to the resin matrix can reduce the surface free energy of the coating [20,21]. Moreover, the incorporation of nano-SiO_2_ particles to the coating with low surface energy can not only construct the micro-rough surface structure, increasing the strength of polymer materials, but also improve the wear resistance and corrosion resistance of the coating [22,23,24]. Shin et al. [25] synthesized a series of waterborne fluorinated acrylate-based PUs for application in antifouling coatings. Krol et al. [26] prepared a kind of hydrophobic WPU coating by the incorporation of fluorine. Serkis et al. [27] prepared a new type of water-based PU/silica nanocomposite by the addition of nano-SiO_2_ particles. Zhang et al. [28] used modified nano-SiO_2_ particles to synthesize UV-curable water-based transparent coating. Generally, the performance of the WPU coating has been improved to some extent by modification, but the process is complex and time-consuming, moreover, the dispersion of nano-fillers has not been well dissolved. Therefore, the method of electrostatic spraying nano-SiO_2_ particles was used in this work to construct micro-rough surface structure on the coating with low surface energy and to improve the properties such as hydrophobicity and wear resistance. Electrostatic spraying is pollution-free and has excellent atomization capability and operability. In addition, nano-SiO_2_ particles can be well dispersed under the action of a high-voltage electrostatic field and most importantly, the driving force arising from the compressed air may make the nanoparticles greatly adhere to the coating surface [29].

The modified PTFE particles with different contents were added to the WPU dispersions to prepare WPU composites with low surface energy (LSWPUCs). Then, nano-SiO_2_ particles were sprayed using an electrostatic spraying device on the surfaces of the uncured LSWPUCs to form a micro-rough surface in this work. Thus, the superhydrophobic WPU composites (SHWPUCs) were formed owing to the adhesion of nano-SiO_2_ particles in the coating during curing. The adhesion, corrosion resistance and wear resistance of the obtained SHWPUCs were measured to evaluate their durability. Additionally, the effects of the micro-rough surface structure and the hydrophobic system content on the properties of WPU composites were studied. This may provide a useful reference for the design, preparation and application of superhydrophobic waterborne coatings.

## 2. Materials and Methods

### 2.1. Experimental Materials

WPU dispersion was supplied by Jining Huakai Resin Co. Ltd. (Jining, China). Its solid content, viscosity and VOC concentration were 35%, 75 cps and 253 g/L, respectively. Polydimethylsiloxane (PDMS) emulsion (average *M*_w_ = 115,000 g mol^−1^) was supplied by Shanghai Yuanye Biotechnology Co. Ltd. (Shanghai, China). Ethyl silicate (TEOS, analytically pure) was supplied by Tianjin Kermio Chemical Reagent Co. Ltd. (Tianjin, China). Dioctyldilauryltin (DOTDL, *M*_w_ = 743.7 g mol^−1^), 98%, was supplied by Shanghai Yuanye Biotechnology Co. Ltd. (Shanghai, China). Sodium chloride (NaCl, analytically pure) was supplied by Tianjin Kemio Chemical Reagent Co. Ltd. (Tianjin, China). Anhydrous ethanol (analytically pure) was supplied by Tian in Fuyu Fine Chemical Co. Ltd. (Tianjin, China). PTFE particles (MP1200) particles with average particle size ≤3 µm were purchased from Dupont (Wilmington, DE, US). Nano-SiO_2_ (N-100) particles were supplied by Jining Huakai Resin Co. Ltd. (Jining, China). The average diameter, specific surface area, and silica content of nano-SiO_2_ were respectively 40–60 nm, 300 ± 25 m^2^/g and 99.8%, respectively.

Q235 steel (50 mm × 20 mm × 3 mm) was used as the metal substrate and was roughened by a YX-6050A sand blasting device (Anbangruiyuxin Machine Technology Development Co. Ltd., Wuhan, China). The air pressure was 0.6–0.8 MPa. The distance between the Q235 steel substrate and the spray gun was 110–150 mm. The spray time was 30–40 s.

### 2.2. Preparation of the LSWPUCs

**Figure 1:**
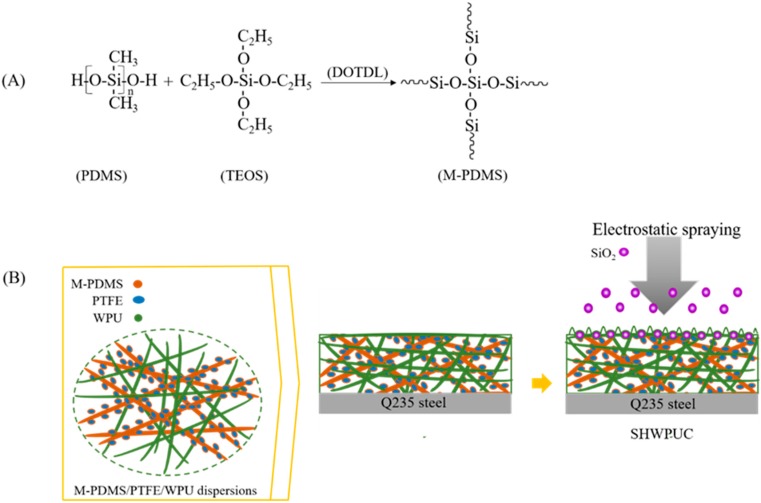
(**A**) modification of the PDMS emulsion and (**B**) Schematic illustration of the superhydrophobic WPU composite (SHWPUC).

PTFE particles should be modified to improve the dispersibility before adding. At room temperature, PDMS, TEOS (5 wt % vs. PDMS) and DOTDL (1.5 wt % vs. PDMS) were mixed by an 85–2 magnetic stirring device (Hangzhou Instrument Motor Co., Ltd., Hangzhou, China) at a speed of 100–150 r/min for 30 min. Then, the mixture was treated by a KQ-50B ultrasonic dispersion device (Kunshan Ultrasonic Instrument Co., Ltd., Kunshan, China) for 10 min to prepare the modified PDMS (M-PDMS) emulsion (shown in Figure 1A). Subsequently, the M-PDMS emulsion and PTFE particles were added to WPU by magnetic stirring at a speed of 250–300 r/min for 40 min. The obtained M-PDMS/PTFE/WPU dispersions (shown in Table 1) were first placed at room temperature for 20–30 min and then coated on the Q235 steel substrates. The thickness of the coatings (110–120 mm) was controlled by weighting during coating. After coating, the samples were cured first at room temperature for 1 day and then at 150 °C for 1 h in an oven (Zhejiang Yuyao Yuandong CNC Instrument Factory, Yuyao, China). Thus, a layer of the LSWPUC was prepared on the Q235 steel substrate. The samples were named as #1 LSWPUC, #2 LSWPUC, #3 LSWPUC and #4 LSWPUC. In addition, the pure WPU coating (named as #0) was also cured in the same way described previously for reference.

### 2.3. Preparation of the SHWPUCs

The M-PDMS/PTFE/WPU dispersions were prepared and then coated on the Q235 steel substrates in the same way described previously. After coating, the samples were placed at room temperature for 2–3 h. In this case, the obtained coatings were under semi-dry and nonflowing conditions and their surfaces were slightly tacky. In order to enhance the dispersion and adhesion of nano-SiO_2_ particles, a NEW KCI-CU801 electrostatic spraying device (Shenzhen Honghaida Instrument Co., Ltd., Shenzhen, China) was used to spray SiO_2_ nanoparticles on the uncured coating surfaces to construct the micro-rough surface structure. The sprayed content of nano-SiO_2_ particles was 10 g/m^2^. The voltage of electrostatic spraying was 50–60 kV. The pressure of the compressed air was 0.6–0.7 MPa. The spraying time was 20–30 s. The distance between the spray gun and the sample was 110–150 mm. After spraying, the samples were cured first at room temperature for 1 day and then at 150 °C for 1 h in an oven. Thus, a layer of the SHWPUC was completely prepared on the Q235 steel substrate. The schematic illustration of the LSWPUC is shown in Figure 1. The obtained samples were named as #1 SHWPUC, #2 SHWPUC, #3 SHWPUC and #4 SHWPUC.

### 2.4. Measurements

#### 2.4.1. WCA Test

The WCAs of the coatings were measured by an SDC-200 contact angle testing machine (Dongguan Shengding Precision Instrument Co., Ltd., Guangzhou, China) to characterize the hydrophobicity. A 20 µL pure water droplet was injected with a micro syringe for each test. Each of the two samples for each coating was measured six times and the measurements were averaged to determine the WCA.

#### 2.4.2. Adhesion Test

The adhesion test was performed according to ISO 4624:1978 at room temperature by a D2-5DL universal mechanical testing machine (Changchun Mechanical Institute, Changchun, China). The coating was totally peeled off from the steel substrate during test. Each coating was measured ten times and the measurements were averaged to calculate the adhesion by Equation (1):

(1) σ=P/A,

where σ is the adhesion (MP), *P* is the maximum load (N) and *A* is the coating area (mm^2^).

#### 2.4.3. Corrosion Resistance Test

A 3.5 wt % NaCl solution was used as the corrosion medium to test the corrosion resistance of the obtained coatings under harsh conditions. The polarization curve and electrochemical impedance spectroscopy of the coating were measured at room temperature by a Ver4.2corr Test System (Wuhan Corr Test Co., Ltd., Wuhan, China) with a three-electrode cell. The sample was used as the working electrode, the Pt electrode was used as the auxiliary electrode, and the saturated calomel electrode was used as the reference electrode. The test area was 0.785 cm^2^. The samples were immersed in 3.5 wt % NaCl solution at 40 °C for 30 days before testing. The testing was started when the open circuit potential of the system was stable.

#### 2.4.4. Wear Resistance Test

The wear resistance test of the coating was carried out at room temperature in accordance with ASTM G99-05 by an HT-1000 high temperature scratch testing machine (Lanzhou Zhongke Kaihua Development Co., Ltd., Lanzhou, China), with the coating against a steel bearing ball (Ø2.5 mm) with a hardness level of HRC62. The applied load was 5 N, the rotation speed of the steel ball was 400 r/min, the sliding radius was 5 mm, and the wear time was 10 min. The wear rate and friction coefficient were used to evaluate the wear resistance of the coating. The wear rate was calculated by Equation (2).

(2) I=Δm/2πrntFρ,

where *I* is the specific wear rate (cm^3^/mm N), Δ*m* is the loss weight (g), *r* is the sliding radius (mm), *n* is the rotation speed of the steel ball (r/min), *t* is the wear time (min), *F* is the applied load (N) and ρ is the density of the WPU coating (g/cm^3^).

#### 2.4.5. Morphology Analysis

The 3D micromorphologies of the coating surfaces were observed by an LEXT OLS4000 laser confocal scanning microscope (OLYMPUS, Tokyo, Japan) to characterize the effect of the micro-rough surface structure on the hydrophobicity of the coatings. The magnification was 108–17,280×; the field of view was 16 µm to 2.56 mm.

After the wear test, the surface morphologies of the wear tracks were observed by a VEGA3 XMU Scanning Electron Microscope (SEM) (TESCAN, Brno, Czech).

## 3. Results and Discussion

### 3.1. Hydrophobicity of WPU Composites

**Figure 2:**
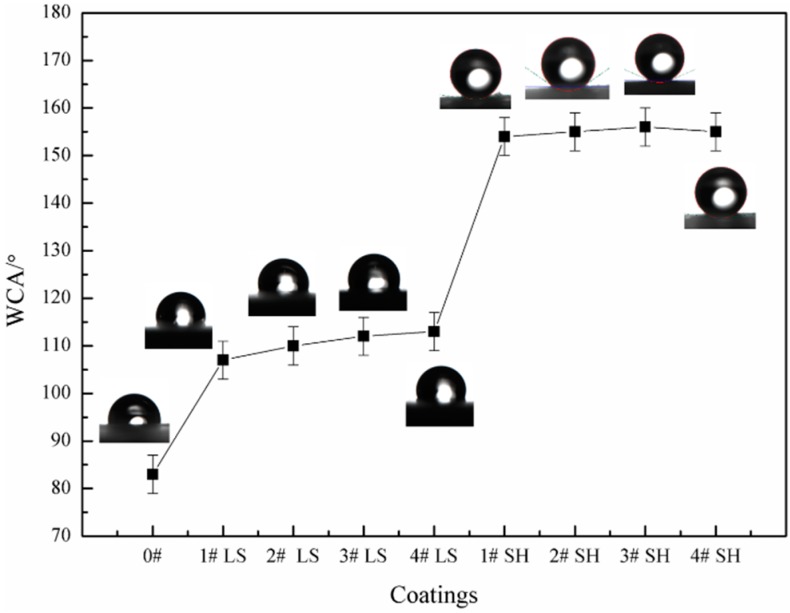
Water contact angles (WCAs) of the WPU composites with different M-PDMS/PTFE system contents.

Figure 2 shows the WCAs of different WPU composites. The WCAs of #0 (pure WPU coating), #1 LSWPUC, #2 LSWPUC, #3 LSWPUC and #4 LSWPUC were 83°, 107°, 110°, 112° and 113°, respectively, stating that the low surface energy coating was successfully prepared by the incorporation of the hydrophobic system of M-PDMS/PTFE. When the M-PDMS/PTFE system content was below 8.05 wt % (#3 M-PDMS/PTFE system vs. M-PDMS/PTFE/WPU system), the WCAs of the LSWPUCs first increased and then remained stationary as the M-PDMS/PTFE system content rose. The reason may be as follows. PTFE as a kind of symmetrical and non-polar polymer has high fluoride content and low surface energy. PDMS holds low surface energy and the M-PDMS will generate a crosslinked network structure after modification. Then, the M-PDMS as the modifier in this work would adsorb on the surfaces of PTFE particles. Under the action of magnetic stirring, the M-PDMS/PTFE system could be evenly dispersed in the WPU dispersion, leading to the reduction of the surface energy of the WPU composites. When the M-PDMS/PTFE system content reached 8.05 wt %, the hydrophobic groups of the coating surface were under the saturated condition. As the M-PDMS/PTFE system content continued to increase, the change in the WCA of the LSWPUC remained stable.

It can be seen from Figure 2 that the WCAs of #1 SHWPUC, #2 SHWPUC, #3 SHWPUC and #4 SHWPUC were 154°, 155°, 156° and 155°, respectively, displaying their superhydrophobic behaviors. This indicated that electrostatic spraying nano-SiO_2_ particles on the coating surface with low surface energy could construct the micro-rough surface structure and then improve the hydrophobicity of the coating. This may be explained by the following. The WPU composites were all in the uncured state when nanoparticles were sprayed. Under the action of high-voltage electrostatic field, nano-SiO_2_ particles would first enter the locations of the coatings where there were some defects or less nano-SiO_2_ particles and thus result in an even distribution. In addition, nano-SiO_2_ particles can be rushed into the uncured composites due to the high air pressure of electrostatic spraying. Subsequently, a layer of uniform WPU composites could be coated on the surfaces of nano-SiO_2_ particles [23]. Therefore, the micro-rough surface structure was formed on the surfaces of the WPU composites, as shown in Figure 1.

**Figure 3:**
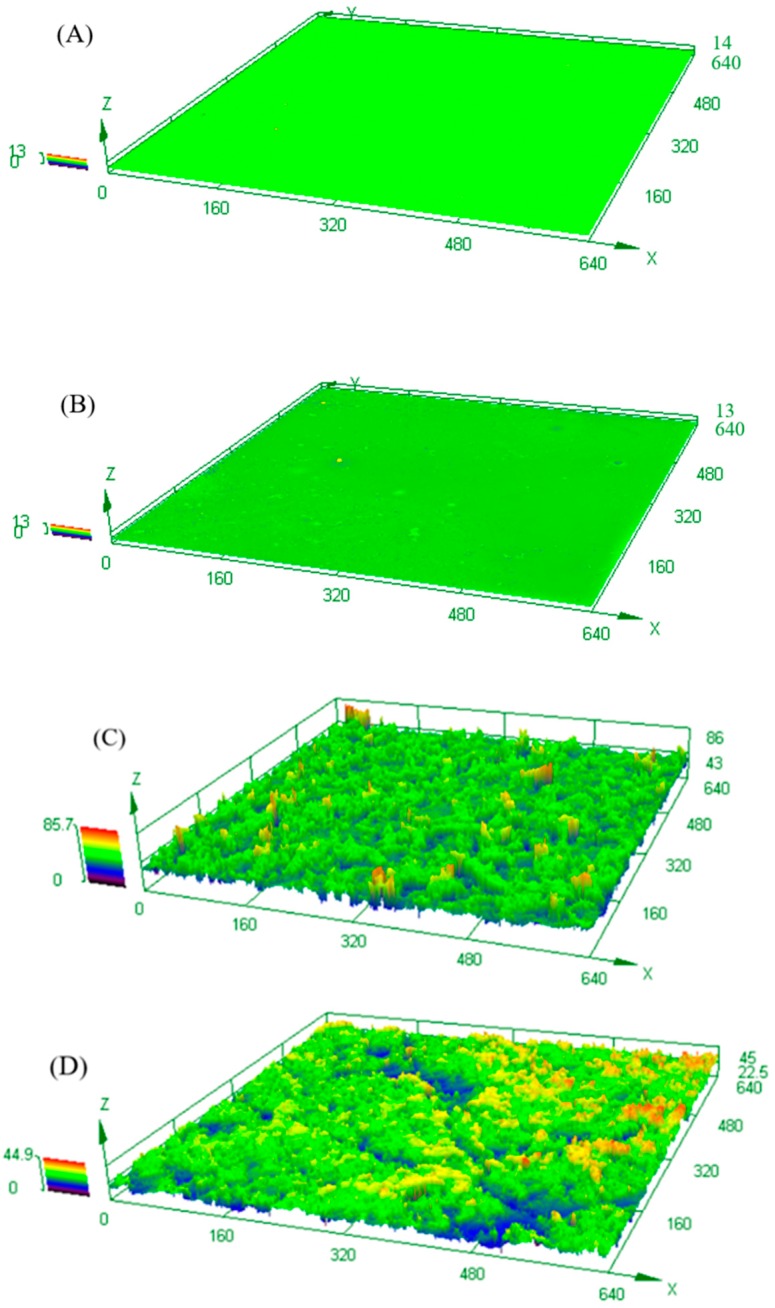
Micromorphologies of different WPU composites of (**A**) #0; (**B**)#3 LSWPUC; (**C**)#3 SHWPUC; and (**D**) #4 SHWPUC, (µm).

Figure 3 shows the 3D micromorphologies of different coating surfaces as observed by laser confocal scanning microscope. The surface micromorphology of #0 (Figure 3A) was as smooth as that of #3 LSWPUC (Figure 3B), indicating that the incorporation of M-PDMS/PTFE system would not greatly affect the surface micromorphologies of the prepared WPU composites. The micro-rough surface structure on the surface of #3 SHWPUC (Figure 3C) was similar to that of #4 SHWPUC (Figure 3D). However, due to the facts that a low content of the M-PDMS/PTFE system was added to #3 SHWPUC and that the number of nano-SiO_2_ particles embedded in its surface was relatively uniformly compact, the resulting micro-rough structure of #3 SHWPUC was a little better than that of #4 SHWPUC. This result was consistent with that of the WCA tests. Moreover, it demonstrated that the hydrophobicity of the coating may be closely related to the micro-rough surface structure and the superhydrophobic coating can be prepared by electrostatic spraying [30].

According to the Wenzel equation, $\cos\theta_{\omega }=r\cos\theta_{0}$, where $\theta_{\omega }$ is the apparent contact angle, $\theta_{0}$ is the eigen contact angle and r is the micro-roughness. When $\theta_{0}$ of the coating was over 90°, its hydrophobicity increased as the surface micro-roughness rose. It can be seen that the LSWPUCs prepared by the addition of the M-PDMS/PTFE system to WPU were hydrophobic; their WCAs were over 90°. In addition, spraying nano-SiO_2_ particles on the coating surfaces increased the micro-roughness and obtained the SHWPUCs whose WCAs were greater than 150°.

In the case of Cassie–Baxter state, the rough surface is regarded as a compound interface composed of air and solid. Water droplets can stand at the top of the rough structure of the superhydrophobic surface which has very low adhesion to water droplets. According to Cassie–Baxter equation, $\cos\theta_{c}=f(1+\cos\theta_{0})-1$, where $\theta_{c}$ is the apparent contact angle, $\theta_{0}$ is the eigen contact angle, and f is the solid–liquid contact area percent. $\theta_{c}$ can increase with the decrease of f. As for #3 SHWPUC, its $\theta_{0}$ and $\theta_{c}$ were 112° and 156°, respectively, and it was calculated that f = 0.13. That is, the solid–gas contact area percent reached 87%, indicating that a large amount of gas was between the micro-rough structures of the coating surface and thus the coating was superhydrophobic.

### 3.2. Adhesion of the SHWPUCs

**Figure 4:**
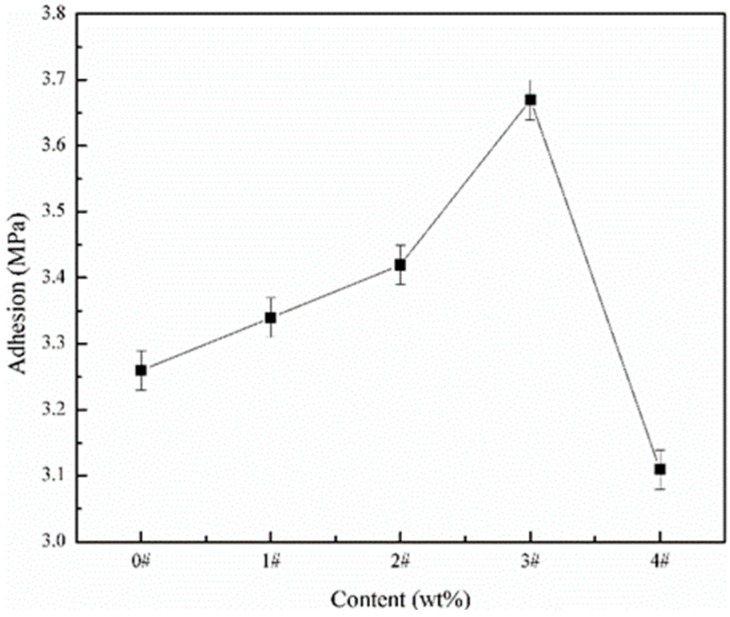
Adhesion of different SHWPUCs to the steel substrates.

The adhesion of the coating to the steel substrate is a main performance evaluation. Figure 4 shows the adhesion of different SHWPUCs to the steel substrates. With an increase in the M-PDMS/PTFE system content, the adhesion of the SHWPUC to the Q235 steel substrate first increased and then declined. The adhesions of #0, #1 SHWPUC, #2 SHWPUC, #3 SHWPUC and #4 SHWPUC were 3.26, 3.34, 3.42, 3.67 and 3.11 MPa, respectively. The adhesion of #3 SHWPUC was the best among all the obtained coatings, which increased by 12.58% compared with that of #0. The reason may be that the addition of a small number of PTFE particles to the WPU resins could fill the pores arising from the water evaporation during curing of WPU and then the contact areas between the WPU resin and the metal substrate could increase. Thus, the adhesion of the coating would be strengthened. Additionally, the pores of #3 SHWPUC were basically filled when the M-PDMS/PTFE system content reached the critical value of 8.05 wt %, so that the coating structure became dense and the adhesion to the steel substrate was enhanced. As the M-PDMS/PTFE system content went on to rise, the excessive PTFE particles would occupy too much contact area between the WPU resins and the metal substrate, leading to the reduction of the adhesion of #4 SHWPUC.

### 3.3. Corrosion Resistance of the SHWPUCs

**Figure 5:**
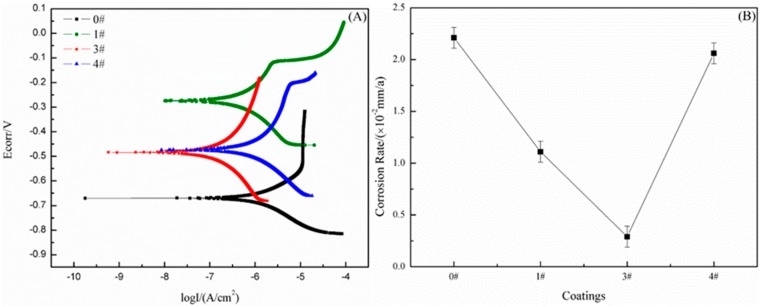
(**A**) Polarization curves and (**B**) corrosion rates of different SHWPUCs immersed in 3.5 wt % NaCl solution at 40 °C for 30 days.

Figure 5A shows polarization curves of the SHWPUCs with different M-PDMS/PTFE system contents after an immersion in 3.5 wt % NaCl solution at 40 °C for 30 days. Figure 5B shows corrosion rates of different SHWPUCs which were calculated by computer software. The corrosion rates of #0, #1 SHWPUC, #3 SHWPUC and #4 SHWPUC were 2.21 × 10^−2^, 1.11 × 10^−2^, 0.29 × 10^−2^ and 2.06 × 10^−2^ mm/a, respectively. As the M-PDMS/PTFE system content increased the corrosion resistance of the SHWPUC first increased and then declined. The facts may be as follows. When the M-PDMS/PTFE system content was low, PTFE particles could fill the pores generated by the water evaporation of the WPU coating during curing. Then, the coating structure would become relatively compact to prevent the diffusion of the corrosive medium. Thus, the corrosion resistance of the coating would be improved. However, with M-PDMS/PTFE system content over 8.05 wt %, the increased interfaces between PTFE particles and WPU resins could increase the diffusion ability of the corrosive medium and thus reduce the corrosion resistance of the coating. The corrosion rate of #3 SHWPUC was about one order of magnitude lower than that of #0. The coating structure and corrosion resistance of #3 SHWPUC were the best among all the obtained coatings.

**Figure 6:**
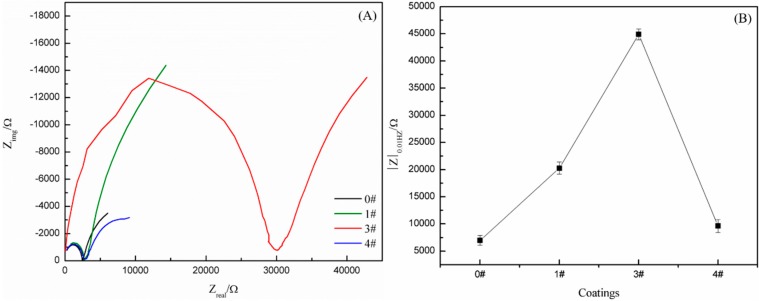
(**A**) Nyquist plots and (**B**) |Z|_0.01Hz_ values of different SHWPUCs after an immersion in 3.5 wt % NaCl solution at 40 °C for 30 days.

Figure 6A shows Nyquist plots of the SHWPUCs with different M-PDMS/PTFE system contents immersed in 3.5 wt % NaCl solution at 40 °C for 30 days. A nearly complete semicircle was observed in the high frequency range on the Nyquist plots and the semicircle diameter showed the insulation and shielding properties of the coating [29]. As the M-PDMS/PTFE system content increased, the semicircle diameter in the high-frequency range of the SHWPUC first increased and then decreased. The semicircle diameters in the high frequency range of #1 SHWPUC, #3 SHWPUC and #4 SHWPUC were all larger than that of #0. Furthermore, the largest semicircle diameter in the high frequency region of #3 SHWPUC would hold the best shielding performance against the corrosive medium.

A quarter semicircle in the low frequency range was related to the corrosion reaction between the electrolyte and the metal substrate and clearly stated that the NaCl solution had penetrated to the Q235 steel substrate after an immersion for 30 days. Figure 6B shows the |Z|_0.01Hz_ values of the SHWPUCs with different M-PDMS/PTFE system contents. The |Z|_0.01Hz_ value of the SHWPUC first increased and then decreased with the increase of the M-PDMS/PTFE system content. The |Z|_0.01Hz_ values of #0, #1 SHWPUC, #3 SHWPUC and #4 SHWPUC were 6,988.47, 20,267.16, 44,888.46 and 9,618.51, respectively. A higher |Z|_0.01Hz_ value of the coating in the low frequency range indicated that the coating may have a stronger shielding effect on the external corrosive medium. The |Z|_0.01Hz_ value of #3 SHWPUC was the highest among all the obtained coatings, which was 75.24% higher than that of #0. The result further indicated that the coating structure of #3 SHWPUC was the densest among all coatings.

### 3.4. Wear Resistance of the SHWPUCs

**Figure 7:**
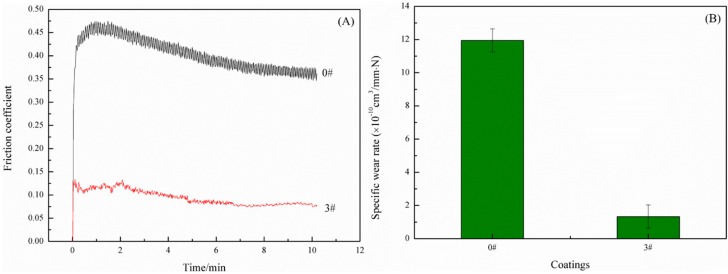
(**A**) Friction coefficient–time curves and (**B**) wear rates of #0 and #3 SHWPUC.

It is evident that the hydrophobicity and corrosion resistance of the obtained #3 SHWPUC were the best among all WPU composites. Therefore, the wear resistance of #3 SHWPUC was further investigated and it was compared with that of the pure WPU coating under same conditions. During the wear test, the applied load was 5 N, the rotation speed of the steel ball was 400 r/min, the sliding radius was 5 mm and the wear time was 10 min. Figure 7A shows the friction coefficient–time curves of #0 and #3 SHWPUC, and Figure 7B shows their wear rates. The friction coefficient–time curve of #0 was uniformly wavy, however, the friction coefficient–time curve of #3 SHWPUC first greatly fluctuated and then became smooth after 6 min. This may be ascribed to the low surface friction of the fluorine element [24]. The wear rate (1.33 × 10^−10^ cm^3^/mm N) and friction coefficient (0.08) of #3 SHWPUC declined by 88.82% and 78.38%, respectively, compared with those of #0, 1.19 × 10^−9^ cm^3^/mm N and 0.37. The reason for this would be that under the action of a high-voltage electrostatic field the surface activity of nano-SiO_2_ particles was probably enhanced and then polar groups such as –OH groups were absorbed on the surfaces of nano-SiO_2_ particles. Thus, the polymerization reaction that occurred between these polar groups and some polar groups in the molecular structure of WPU during curing made nano-SiO_2_ firmly embed to the composite coating, so that it was difficult for nano-SiO_2_ particles to wear off. In addition, the high hardness and strength of nano-SiO_2_ particles could improve the strength of #3 SHWPUC, whose wear resistance was better than that of #0.

**Figure 8:**
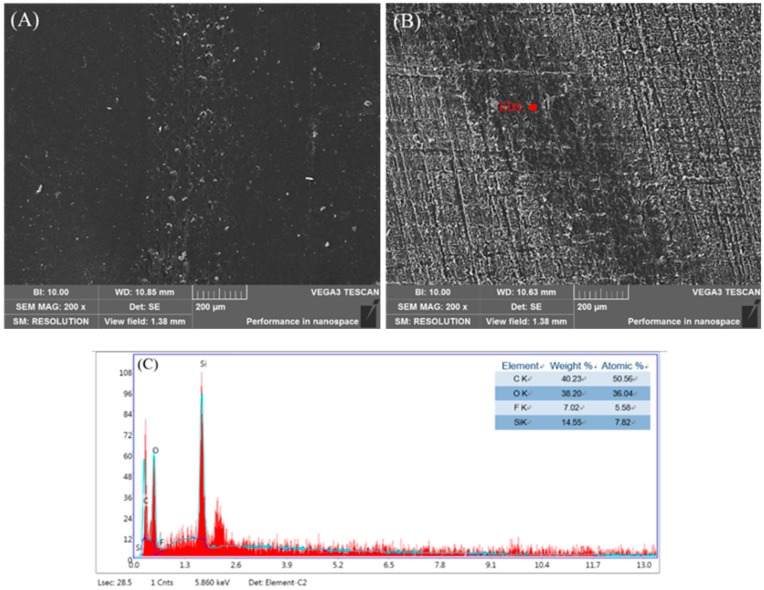
Wear track morphologies of (**A**) #0; and (**B**) #3 SHWPUC. (**C**) EDS of #3 SHWPUC.

Figure 8 shows the wear track morphologies of #0 and #3 SHWPUC after the wear test. There were a few worn pieces on the wear track of #0 (Figure 8A), which would become abrasive materials to exacerbate the wear of the coating. The wear rate and friction coefficient of #0 were aggravated and a wavy friction coefficient–time curve was generated. It is obvious that there were almost no block pieces on the wear track of #3 SHWPUC and its wear track seemed to be shallow (Figure 8B). EDS shows that there were a large number of Si and F elements in the wear track of #3 SHWPUC (Figure 8C), which may have reduced its friction coefficient and wear rate and thus improved its wear resistance. This further indicated that the superhydrophobic surface of the coating with excellent wear resistance can be constructed by electrostatic spraying.

## 4. Conclusions

A series of superhydrophobic WPU composites with micro-rough surface structure were prepared by electrostatic spraying nano-SiO_2_ particles on the obtained WPU composites with low surface energy. It was concluded that the hydrophobic WPU composite was prepared by adding the M-PDMS/PTFE system to the WPU dispersion. As the M-PDMS/PTFE system content rose, the WCAs of the composites first increased and then remained stationary; however, the adhesion and corrosion resistance first increased and then decreased. An appropriate addition of the hydrophobic system content would lead to a dense coating structure, but an excessive addition could increase the interfaces in the coating and then reduce the coating performance. When the mass ratio of the WPU dispersion, PTFE particles and M-PDMS was 8:0.3:0.4, 10 g/m^2^ nano-SiO_2_ particles were sprayed on the uncured coating surface to construct the #3 SHWPUC, whose WCA was 156°. Compared with the pure WPU coating, its adhesion (3.67 MPa) increased by 12.5%, its corrosion rate (0.29 × 10^−2^ mm/a) was reduced by almost one order of magnitude, and moreover, its wear rate (1.33 × 10^−10^ cm^3^/mm N) and friction coefficient (0.08) decreased by 88.8% and 78.3%, respectively.

## Figures and Tables

**Table 1:** System of the modified polydimethylsiloxane (M-PDMS)/polytetrafluoroethylene (PTFE)/waterborne polyurethane (WPU) dispersions.

Samples	#1	#2	#3	#4
WPU (g)	6	8	8	6
PTFE (g)	0.1	0.2	0.3	0.3
M-PDMS (g)	0.2	0.2	0.4	0.6
